# Mothers’ experiences of bonding with their subsequent child following a previous perinatal bereavement

**DOI:** 10.3389/fpsyt.2026.1894993

**Published:** 2026-10-07

**Authors:** Rebecca Hefferman-Clarke, Lynsey Gregg, Lucy Singer, Anja Wittkowski

**Affiliations:** 1 Division of Psychology and Mental Health, School of Health Sciences, Faculty of Biology, Medicine and Health, The University of Manchester, Manchester, United Kingdom; 2 Department of Clinical Psychology and Department of Research and Innovation, Greater Manchester Mental Health NHS Foundation Trust, Manchester, United Kingdom; 3 The Perinatal Mental Health and Parenting (PRIME) Research Unit, Greater Manchester Mental Health NHS Foundation Trust, Research and Development Department, Manchester, United Kingdom; 4 Children and Parents’ Service, Manchester University Foundation Trust, Manchester, United Kingdom; 5 Little Minds Psychology, Manchester, United Kingdom

**Keywords:** attachment, baby, mothers, perinatal death, women

## Abstract

**Introduction:**

Perinatal bereavement can significantly affect a woman’s psychological wellbeing and shape subsequent experiences of pregnancy and motherhood. However, no studies to date have qualitatively explored the impact of different types of perinatal bereavement experiences on bonding with a subsequent baby. Thus, the present study aimed to a) explore the psychological impact a previous perinatal bereavement had on maternal bonding with their subsequent baby (throughout pregnancy and post-birth) and b) identify the psychological and psychosocial support that helped in facilitating this bond.

**Methods:**

In this qualitative study, women who had experienced a perinatal bereavement and had a subsequent child (up to the age of five) were recruited and interviewed. Data were analysed using Reflexive Thematic Analysis.

**Results:**

Seventeen women took part. Two main themes, with five subthemes, were developed from their interview data. Theme 1, *Bracing for loss*, represented how participants’ experiences of subsequent pregnancies were accompanied by a persistent worry of another loss occurring. Theme 1 also explored how participants coped with high levels of worry and how it shaped early experiences of mother-infant bonding. Theme 2, *Navigating motherhood after perinatal bereavement with a subsequent baby*, captured participants’ experiences of reconstructing their family narrative following the birth of a live baby. Bonding with their subsequent baby was considered in conjunction to the lost baby. This theme also focused on social influences that shaped bonding, such as access to support networks and therapy.

**Discussion:**

This study was the first to explore mother-infant bonding following different types of previous perinatal bereavement. Mother-infant bonding following perinatal bereavement appeared to be a dynamic and non-linear process, shaped by worries of a potential future loss, self-preservation strategies, and mothers’ efforts to integrate their identities as a mother to both their lost and living baby. Continuing bonds with their lost baby appeared to support grief integration and increase emotional capacity for bonding with their subsequent baby. The findings from the current study emphasise the need for trauma-informed and relational approaches within perinatal services that support both maternal wellbeing and the development of the mother-infant bond.

## 1 Introduction

Perinatal bereavement refers to the death of a baby during pregnancy or shortly after birth (1). Definitions of perinatal bereavement can vary internationally according to a number of factors, such as gestational age (2). **Table 1** provides a brief overview of the main types and definitions of pregnancy and baby loss used in the United Kingdom (UK). Miscarriage, stillbirth, neonatal death and TfMR are most commonly referred to across academia and bereavement care. It is estimated that millions of women and their partners are affected by these types of losses annually, with reports indicating that there are approximately 23 million miscarriages, 2.6 million stillbirths and 2.3 million neonatal losses worldwide per year (10–13).

**Table 1:** Brief definitions of pregnancy and baby loss terminology.

Type of loss	Brief definition	References
Early miscarriage	Spontaneous loss of a pregnancy during the first 12 completed weeks of gestation.	Tommy’s (3)
Late miscarriage	Spontaneous loss of a pregnancy from 13 weeks and before 24 completed weeks of gestation.	Tommy’s (3)
Ectopic pregnancy	A pregnancy outside the uterine cavity, most commonly in the fallopian tube; it cannot continue as a viable intrauterine pregnancy and can cause serious complications if untreated.	NICE (4)
Pregnancy of unknown location (PUL)	A situation in which there is a positive pregnancy test but no pregnancy is seen inside or outside the uterus on transvaginal ultrasound at the time of assessment.	NICE (4)
Chemical pregnancy	A very early pregnancy loss, usually before 5 weeks, in which pregnancy hormones are detected but the pregnancy ends before it can be visualised on ultrasound.	Tommy’s (3)
Molar pregnancy	A form of gestational trophoblastic disease in which abnormal placental tissue develops after conception; it may be complete or partial and requires specialist follow-up.	Tommy’s (5)
Early elective abortion	An induced ending of a pregnancy in early gestation, including medical abortion up to and including 10 + 0 weeks’ gestation.	NICE (6)
Termination for medical reasons (TfMR)	An intervention that ends a pregnancy because tests indicate a serious fetal condition, or because pregnancy complications pose significant risks to the pregnant person or baby.	Tommy’s (7)
Antepartum stillbirth	The death of a baby before labour and birth, where the baby is born from 24 completed weeks’ gestation showing no signs of life.	Felker et al.: MBRRACE-UK (8)
Intrapartum stillbirth	The death of a baby during labour or birth, where the baby is born from 24 completed weeks’ gestation showing no signs of life.	Felker et al: MBRRACE-UK (8)
Early neonatal death	The death of a liveborn baby under 7 days of age.	Office of National Statistics (9)
Late neonatal death	The death of a liveborn baby from 7 to 27 completed days of age.	Office of National Statistics (9)

Women describe profound emotional reactions following perinatal bereavement, often compounded by societal norms that fail to recognise the magnitude of the loss and of the parenthood of the mother and father (14). Maternal emotional reactions can vary from heightened anxiety and depression (15), post-traumatic stress (16), and complicated grief (17). Most women become pregnant again within 12 to 18 months after their loss (18, 19). Subsequent pregnancies after perinatal bereavement are often experienced as emotionally complex and ambivalent, with women describing both hope for the subsequent pregnancy and fear another loss would occur (20, 21). To manage emotional complexity and ambivalence, mothers may, consciously, or unconsciously, avoid bonding with a subsequent pregnancy as a protection strategy against further loss (22, 23), a process called *emotional cushioning* (20, 22).

A key element of emotional cushioning involves a mother, consciously or unconsciously, restricting her emotional tie with her subsequent foetus and/or pregnancy. A mother’s emotional tie with their baby is more commonly referred to as the mother-infant bond (24). A healthy and strong mother-infant bond, both *in utero* and following birth, leads to better psychological outcomes, promoting greater overall wellbeing in children (25, 26). The active role mother-infant bonding plays in child development has led to it being an important focus of research. Moderating factors involved in mother-infant bonding are multifaceted, with researchers often citing biological and psychological factors, such as oxytocin and maternal mental well-being (27–29). As a mother’s mental health can influence her ability to form a bond with her infant, perinatal mental health services should be aware of potential risk factors that may impact maternal mental wellbeing and provide interventions to support mothers to identify and maintain the mother-infant bond (27).

There is a small, but growing body of qualitative literature exploring how previous perinatal bereavement can shape experiences of bonding with a subsequent pregnancy and/or baby. For example, in their Interpretative Phenomenological Analysis (IPA) study, Campbell-Jackson et al. (30) interviewed seven couples who also had a history of stillbirth and who had recently had another baby. Parents expressed how bonding “*didn’t feel entirely natural*” (p. 7). Similarly, Budak et al. (31) also used IPA to analyse data from email interviews with six mothers following the birth of an infant after a previous stillbirth. Their analysis revealed that mothers described how bonding with their subsequent baby felt like a betrayal to their baby who had died. More recently, in their IPA study, Blocksidge et al. (32) interviewed six couples who had experienced a previous stillbirth. Bonding was described as secondary to parenting and something to *“deal with after*” (p. 6).Although each of these studies referred to potential bonding challenges, neither focused on mother-infant bonding exclusively. As each of these studies explored experiences of pregnancy and parenting live babies, born following a stillbirth, further research is needed to extend these findings by qualitatively exploring mothers’ experiences of *bonding* with a subsequent baby (throughout pregnancy, through to the arrival of a subsequent live baby).

Thus, the present study aimed to a) explore the psychological impact a previous perinatal loss (as outlined in **Table 1**) and subsequent bereavement had on maternal bonding with their subsequent baby, and b) identify the psychological and psychosocial support that helped in facilitating this bond.

## 2 Methods

### 2.1 Design

In this qualitative study, experiences of bonding with a subsequent infant born following a previous perinatal bereavement were explored through individual interviews. Data were analysed using Reflexive Thematic Analysis (RTA; 33, 34).

For transparency and comprehensiveness, we completed the Consolidated Criteria for Reporting Qualitative Research (COREQ) checklist (35; see supplementary materials).

### 2.2 Ethical approval

Ethical approval was obtained from the National Health Service (NHS) Research Ethics Committee (REC), the Health Research Authority [24/NS/0130], and an NHS Trust in the Northwest of England. Experts by experience were consulted during the research design process, in accordance with best practice UK legislative frameworks (36). Following consultations, the study advertisement and interview guide were slightly revised.

### 2.3 Participant inclusion and exclusion criteria

Participants were eligible if they a) were women or birthing people aged 18 years and above, b) had experience of a previous perinatal bereavement (miscarriage, stillbirth, neonatal loss, TfMR, or baby loss in the first two years^1^ of a baby’s life), and c) had subsequently had another child (under five years of age at the time of interview to ensure mothers could recall specific details pertinent to bonding and psychological and psychosocial support needs). Mothers were excluded if they were not proficient in English or unable to provide informed consent.

### 2.4 Recruitment

Convenience sampling was used to recruit a minimum of 12 and a maximum of 20 participants; whilst Braun and Clark (34) do not offer a ‘target’ sample recruitment, this sample size would be considered appropriate for RTA. Furthermore, we considered the depth of interviews and the richness of experiences being shared (37), with attention also being paid to the emergence of new relevant information in interviews and recruitment continuing until few new themes were identified (e.g., 38).

Participants were recruited via two routes, including through a local NHS Perinatal and Trauma Loss Service (called PETALS). Clinicians identified eligible participants from their caseloads and approached them with the study advertisement. If interested, the potential participant completed a consent to contact form. Study advertisement posters were also shared via social media platforms (Facebook and Instagram) by pages dedicated to supporting mothers following loss. Interested women or birthing people were invited to contact the first author via email.

Brief screening video calls were organised with all potential participants to outline study procedures and ethical considerations and assess eligibility. Screening calls also allowed the interviewer to establish a relationship with participants ahead of the interviews and share details of the purpose for carrying out the research. A participant information sheet was provided to potential participants and follow up contact was arranged 24 hours after the screening call. On gaining consent to take part, a time and date for the interview to be completed either in person or online was scheduled based on the participant’s preference. Informed consent was audio-recorded prior to data collection using an encrypted audio device. Interviews were expected to last between 60 to 90 minutes.

### 2.5 Interview guide development

During the study design, terminology was discussed and considered carefully. For example, the authors discussed the use of the term bonding, paying attention to how it has often been used interchangeably with attachment in the literature (24). To correctly and clearly operationalise bonding for the present study, the most widely accepted definition was used, which refers to a mother and her perception of her own emotional connection towards her pregnancy and/or baby. Whilst it is accepted that bonding mainly refers to the affective state of the mother towards the infant (24), it was important to utilise questions in the interview guide that emphasised behavioural components and that might aid bonding to allow participants to naturally explore this topic further.

An interview schedule with three main areas was developed, based on the available literature and professional experience amongst the research team. This schedule was then used as a topic guide to explore these three main topics. Language was refined based on feedback from Experts by Experience. Topic 1 related to the participants’ experiences of their previous loss. Topic 2 focused on exploring mothers’ experiences of bonding with the child born following their perinatal bereavement and topic 3 allowed mothers to discuss psychological and psychosocial support they received or would have liked to receive to facilitate the bond. Topic areas were a guide and hence used flexibly throughout the interview. The interview guide is available in supplementary materials.

### 2.6 Data collection

Interviews were conducted online via Microsoft Teams and in person, based on participant preference, between March and November 2025. Demographic information (e.g., the participant’s age, loss experience and age of subsequent child) was collected prior to the start of the interview. Interviews were recorded on an encrypted audio device. Upon interview completion, participants were debriefed and offered a few minutes to reflect on the interview content and ask questions. All participants received a £15 shopping voucher as reimbursement for taking part.

### 2.7 Data analysis

All interview data were transcribed verbatim by the first author and stored in a password-protected Word document file. Identifiable information was removed from the data set during this process, and each finalised transcript was checked for anonymity by the first author. Transcripts were then uploaded to NVivo software (NVivo-15) to form part of the overall dataset. Interview data were analysed using RTA (33, 34) by the first author and the process was guided and supervised by the second author. Braun and Clarke’s (33, 39) six phases of RTA were undertaken as follows:

1. Data familiarisation: The first author completed data collection, transcription of all interviews, and data cleaning (checking for accuracy and anonymity). Finalised transcripts were read in full several times (the first five transcripts were also read several times by the second author). The first author also wrote brief summaries for each dataset.
2. Generating initial codes: The first and second author individually completed line-by-line coding for the first five transcripts. Memos and annotations of areas of interest were noted and team discussions on initial codes were held to check for accuracy. The first author completed the coding of the remaining transcripts.
3. Generating initial themes: Data extracts within codes were inspected to assess for broad patterns of meaning across the dataset. Codes were condensed and combined to form initial themes based on the studies research question and aims. Initial codes were discussed in research team meetings with all authors.
4. Reviewing themes: Transcripts were revisited to ensure that the themes aligned with the data. Themes were solidified, merged or discarded.
5. Defining and naming themes: Short descriptions of each theme were written, and themes were named based on overriding features of each theme. Initial themes were drafted and shared with all authors. All authors provided feedback on the themes and the final themes and their subthemes were agreed.
6. Producing a report: The final analysis was written, and the most relevant participant quotations were selected.

### 2.8 Theoretical position

All authors were women with personal and/or professional experiences of perinatal bereavement. The first author was a trainee clinical psychologist who had gained experience in research evaluating trauma-focused interventions for clinical populations in healthcare and academic settings, prior to clinical training. The first author had several years’ worth of experience working clinically in mental health services, delivering psychological interventions in inpatient and community settings. At the time of analysis and thesis submission, she was working therapeutically with cared for children and their carers, using models of attachment. The second, third and fourth authors were all mothers and had clinical and/or research experiences in the field of maternity and perinatal mental health as practising and/or academic psychologists, including working in NHS Children and Parent Service (third author) and a NHS Mother and Baby Unit and specialist perinatal mental health settings (fourth author).

Braun and Clarke (40) view

“researcher subjectivity as a resource” (p.20; 41)

and hence they do not suggest that researcher bias should be eliminated (42). Consequently, the first author kept a research reflective diary to capture her assumptions and biases throughout the study, and she regularly discussed her evolving position as an ‘insider-outsider’ (43) in supervision. Additionally, research supervision and reflective practice groups in clinical training were utilised to consider the emotionality involved in researching a sensitive topic, particularly being a woman of childbearing age.

A critical realist ontology was adopted for this study. Critical realism allowed the researchers to acknowledge that participants’ experiences were constructed by causal psychological mechanisms (44). Causal mechanisms are unseen phenomena that influence the outcome of events (45) and by discovering these causal mechanisms researchers can offer effective guidance. Adopting this approach, alongside RTA, the researchers acknowledged that their prior experience and knowledge shaped the research process and outcomes.

## 3 Results

### 3.1 Participant characteristics

Nineteen potential participants approached the first author expressing interest to take part. All potential participants who took part in the initial screening calls were allocated a unique participant identification number, starting with the letter P for participant, based on the chronology in which they presented to the study. For example, the first participant to attend a screening call was given the identifier P1 and so on. As two participants decided not to take part in the study for personal reasons, 17 participants were recruited. Most participants opted to be interviewed online using Microsoft Teams (n = 15) whilst two wished to be interviewed at their home. Interviews lasted between 50 to 100 minutes (Mean = 77.94 minutes, Standard Deviation = 16.11).

All participants identified as women aged between 29 and 46 years old (Mean = 36.0, Standard Deviation = 4.50). Fifteen of the 17 participants identified as white (i.e., English, Welsh, Scottish, Irish, British, Irish traveller, or any other white background), whilst one identified as ‘mixed/multiple ethnic groups’, and one as ‘other ethnic group’. Participants reported experiences of miscarriage (n=8), stillbirth (n=6), TfMR, (n=4) and neonatal loss (n=3). Other experiences reported by participants also included unsuccessful IVF (n=3) and molar pregnancy (n=1). Nine participants in the study reported more than one type of loss. All losses occurred in earlier perinatal stages (i.e., none occurred after the first 28 days of life). The age range of children born following previous loss was two months to five years old (M = 21.2 months, SD = 21.5 months). Participants lived in different areas of the UK, including the Northwest England (n = 11), East of England (n = 1), East Midlands (n = 1), Southwest England (n = 3) and London (n = 1).

### 3.2 Findings

Following analysis of the 17 interviews, two main themes were developed: Theme 1, *Bracing for loss*, consisted of three subthemes while Theme 2, *Navigating motherhood after perinatal bereavement with a subsequent baby*, consisted of two subthemes. Both themes outline participants’ experiences throughout motherhood (throughout pregnancy, through to the arrival of a subsequent live baby).

**Figure 1** shows a conceptual model of the two main themes and demonstrates how, whilst distinct, Theme 1 and Theme 2 are seen as connected through the influence each might have on the development of the mother-infant bond. The figure illustrates how participants’ experiences of bonding with their subsequent baby appeared to have been shaped by a dynamic interaction between perceived threat, self-protective coping, maternal confidence, family narrative, and support. The arrows indicate how some of the sub-themes appeared to maintain the participants attempts to brace for loss, whereas others appeared to strengthen their ability to bond with their subsequent baby. Together, the themes suggest that bonding after perinatal bereavement was experienced as an evolving and relational process - a non-linear process shaped both by efforts to brace for another loss and by attempts to make sense of motherhood after bereavement.

**Figure 1:**
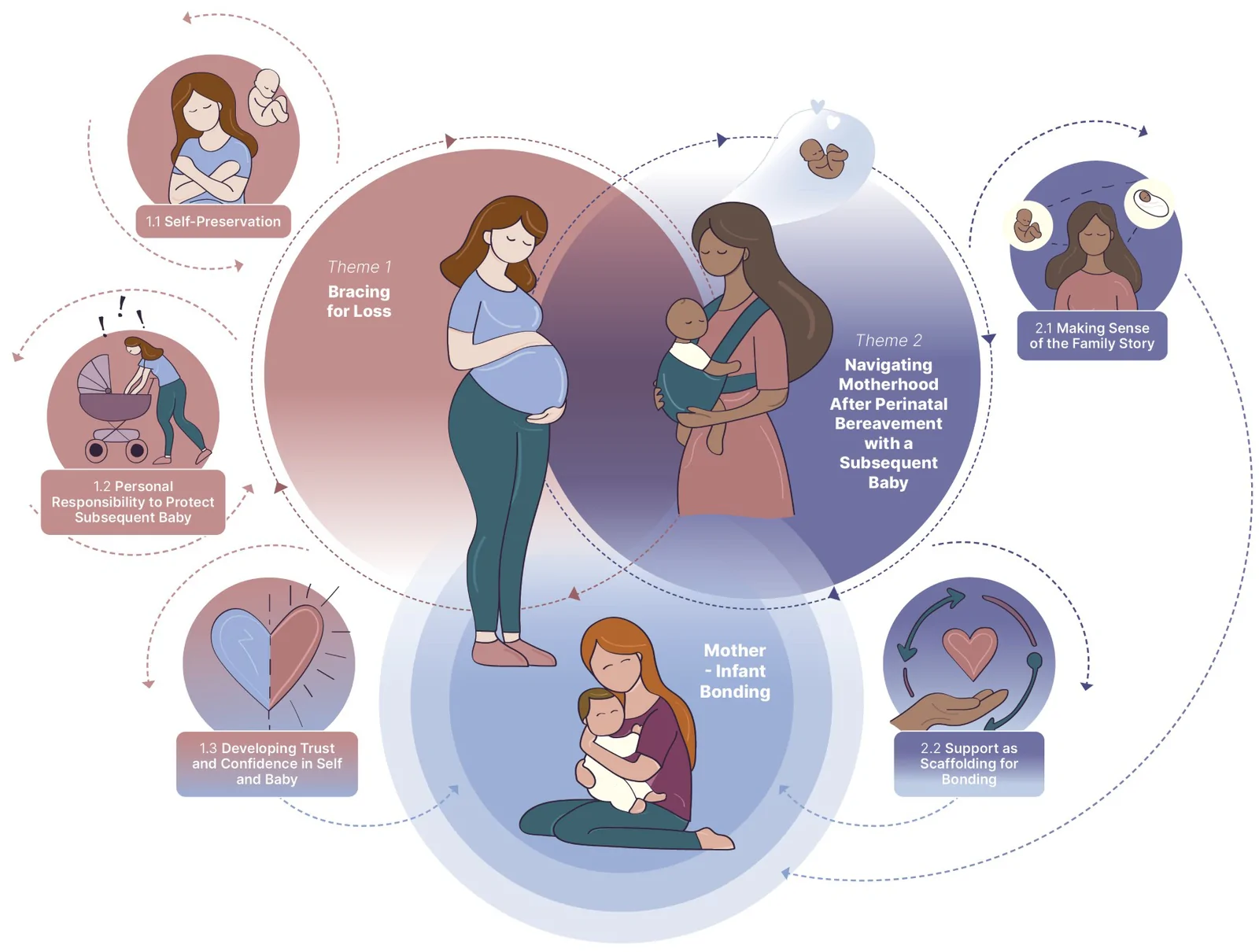
Conceptual diagram of the study’s two main themes and their subthemes.

#### 3.2.1 Theme 1: Bracing for loss

Theme 1 described a persistent sense of perceived threat experienced by participants throughout the subsequent pregnancy and, for some, following the birth of a live baby. Throughout subsequent pregnancies, participants were reminded of the challenges experienced during previous loss which brought attention to potential pregnancy complications. Participants expressed feeling an underlying worry that future loss was imminent which was accompanied by a hypervigilance towards any perceived threat indicative of another potential perinatal bereavement. Threat was a continuous feature across accounts and reflected in a tendency to *brace for loss*. Theme 1 contains three subthemes that outlined how participants coped with this threat perception associated with a worry another loss could occur: 1) *Self-preservation, 2) Personal responsibility to protect subsequent baby, and 3) Developing trust and confidence in self and baby*.

##### 3.2.1.1 Subtheme 1.1: Self-preservation

After the experience of their perinatal bereavement, participants appeared to view their subsequent pregnancy through the context of their previous loss. Participants were more aware of complications that could arise and experienced the subsequent pregnancy as a period of juxtaposing reactions. On the one hand participants were relieved and thankful to be pregnant again, but on the other hand they expressed feeling guilt for the baby that they lost and feared another perinatal bereavement could occur:

“I remember seeing that positive pregnancy test and being such conflicted feelings because you’re excited, but you also feel a dread. Because it is very much like, just because I see a pregnancy, I see a pregnancy ahead, it doesn’t mean we have a baby” (P5).

Activities or experiences that mirrored participants’ last pregnancy, such as attending hospital appointments, were seen to trigger thoughts and feelings related to their loss. Ongoing worries were associated with high levels of anxiety regarding the survival of their subsequent baby throughout the pregnancy and, for some, following birth. Some participants expressed feeling emotionally detached and interactions with their foetus during pregnancy and subsequent baby were procedural rather than emotionally connected. Narratives indicated that the focus on their baby’s survival dampened their experiences bonding with their subsequent baby. In addition, participants outlined how their thinking was dominated by ensuring their foetus’ basic needs were met:

“I just felt anything that acknowledged almost being pregnant bar the absolute critical, essential things that we needed to do, i.e. getting a scan, you know, things that I knew had to happen for my safety and the baby’s safety” (P19).

Participant narratives revealed that thoughts and emotions associated with the possibility of having a viable pregnancy were mentally dampened or blocked altogether because the idea of this pregnancy ending with a live baby felt unrealistic to them at the time:

“Had you asked me that question when I was pregnant, I would have told you that I had bonded with my baby in my bump, but really I hadn’t [ … ] It was just a bump. It was another picture on the screen. It was a graph” (P8).

For some, this emotional avoidance was a conscious protection strategy, highlighting how the experience of a previous perinatal bereavement manifested in the subsequent pregnancy.

Narratives revealed that pregnancies following loss appeared different to participants compared to earlier pregnancies. Participants were less motivated to engage in activities to promote the mother-infant bond, such as speaking to the *bump*, taking *bump* photos and attending classes. Engagement in activities that encouraged bonding were avoided altogether or completed out of fear as opposed to the excitement and joy these activities had brought previously:

“Obviously, like I had times where I’d put my hand on my belly, but generally it was putting my hand on my belly saying ‘please will you survive? Please will you live?’ It wasn’t like how it was with [older child] where I was like, “oh hello baby!” and reading books and things like that. [ … ] it would be me talking to her going, ‘please, please survive’ “ (P19).

In contrast, some participants were motivated to bond with their foetus during pregnancy because they believed they had

“to enjoy this, as this may be the only bit we get” (P4).

The contrast between participants’ approach to forming connections with their foetus throughout pregnancy exemplified the complexity of mother-infant bonding during subsequent pregnancies. Previous loss was a key feature in the development of the mother-infant bond, unconsciously or consciously, and ultimately driven by a perceived threat that another loss would occur imminently.

##### 3.2.1.2 Subtheme 1.2: Personal responsibility to protect subsequent baby

The transition of becoming a mother to a living baby following a previous loss was encompassed by an acute sense of personal responsibility to be the protector of their subsequent baby to ensure survival. For many, limiting emotional investment throughout the pregnancy due to anticipatory loss meant that the arrival of a live baby was met with a sense of shock and disbelief and was accompanied by an initial apprehension about their own ability to care for a live baby:

“I’d researched everything that could go wrong in pregnancy and everything that could go wrong in labour, I hadn’t thought ahead as to ‘what do I do when the baby’s here?’ “ (P8).

Compounding these feelings was the challenging journey following perinatal bereavement participants had been on to give birth to a live baby, some participants were left feeling doubtful, thinking

“this is all I ever wanted, now I don’t know what I’m doing” (P8).

The previous loss appeared to be core to participants’ subsequent experiences of pregnancy and bonding. The ability to bond and form connections was overshadowed by a personal responsibility to be the protector of the subsequent baby. Participants with older children reflected on changes in parenting styles following the loss of their baby:

“With [subsequent baby], she’s been with nobody. So [older child] stayed out from being a few months old, [subsequent baby] I barely left her with my husband” (P19).

Referring to who was involved in the early stages of caring for an older child, one participant described how it felt

“like a village” (P9).

Initially, the reported change in their approach to parenting could be interpreted as a lack of trust in others. However, participants described how their heightened anxiety, sense of possible threat, and pressure to be protector shaped how they cared for, and connected with, their babies born after a previous loss. Narratives revealed that worries had become internalised and, for some, internalisation of worries was echoed in negative self-appraisals of themselves as a mother. For instance, not being

“cut out for motherhood” (P15)

or feeling like they were inevitably

“going to fail [ … ] do something wrong” (P3).

Some participants described trying to reduce their anxiety by engaging in repeated checking behaviours, asking for reassurance from others and trying to prevent anything that could potentially harm their baby. Attempts to manage their fears served to provide brief moments of relief from anxiety; however, over time, behavioural strategies maintained anxieties and reinforced participants’ beliefs that they were the protector:

“Because of suffering with PTSD, I had it in my head, as soon as we come home with [subsequent baby] [ … ], something bad was going to happen. Or that there was something wrong with her. [ … ] So like I was just constantly on edge. I couldn’t enjoy her. It was just surviving, basically, and just keeping her fed, keeping her clean, like making sure she’s sleeping, that sort of thing” (P14).

Interestingly, one participant experienced a decrease in their sense of personal responsibility following the birth of a live baby:

“Once he’s out of me, it’s not all my responsibility to keep him alive. [ … ] it’s not just me, you know, there’s other people that can look after him and take care of him as well. It’s not all on my shoulders” (P5).

The relief experienced in this quotation echoed the personal pressure and responsibility felt by participants to maintain their subsequent baby’s survival.

##### 3.2.1.3 Subtheme 1.3: Developing trust and confidence in self and baby

Confidence in their parenting abilities was a reoccurring feature in the development of the mother-infant bond; not only through the mother’s growing confidence to trust in her own abilities to care for their subsequent baby, but also through noticing a naturally developing reciprocal relationship between the self and baby:

“The sort of bonding aspect for me definitely started probably by the time we were starting to smile and stuff like that. I think I could see that as a mum I was having that positive effect on him. And I think that’s when the penny dropped that, like, ‘right, I’m his mum now and I’m his world and I’ve a) got to enjoy it and b) not feel too guilty for other babies’. But also, you know he needs me and I want to be able, able to give, give him that. So I think that’s when I started to really connect with him. It wasn’t necessarily an immediate ‘he’s born, he’s here’. [ … ] whether you’ve lost babies or not, sometimes it’s not that immediate thing, but I think with him it definitely took a little bit longer and I would put that down to to losing [lost baby]” (P11).

Witnessing their subsequent babies reach developmental milestones was accompanied by an increase in their confidence in their approach to parenting. Participants’ perceptions of being a protector of their subsequent baby were no longer connected to a worry of loss, but they were described now as intertwined with a newfound confidence in their abilities to be a provider and meet their subsequent baby’s needs:

“I loved it when she put weight on and got bigger, it made me more confident because I could see the results of her feeding and stuff. So with me that kind of bond and confidence got stronger because I felt like she knows me and trusts me now and I’m the same with her” (P3).

Trust and confidence in oneself seemed to regulate participants’ worry and was often accompanied by a greater emotional availability to focus on strengthening their bond with their subsequent baby. Some participants reflected on a change in their approach to parenting. Participants were able to allow their baby to explore and become more independent as they grew older:

“I want to be with him and looking after him and taking care of him. And sometimes that’s where, like as he’s growing up, I have to pull myself back because I don’t want to, you know, swamp him too much if you know what I mean. You know, like I want him to be able be independent and be confident” (P5).

Similarly, participants who sought assistance from mental health services to support their own wellbeing and develop the mother-infant bond reported parallel processes. According to the narratives, support guided participants to intentionally notice times of bonding with their baby which shaped the participant’s confidence and trust in herself. This finding presented a positive cycle whereby an increase in self-confidence and trust was linked to participants’ ability to emotionally self-regulate and ultimately appeared to shape bonding experiences:

“I’d look at [another mother and baby] relationship, the way they spoke to each [other], like spoke to their children, the way they, you know their children would look at them. I just didn’t notice that with me and [subsequent baby]. I just, I just didn’t see that in us. But obviously, once the perinatal team pointed it out and showed me everything, it started to slowly, like gradually I was like, ‘oh, I I can, you know, see what they mean now’ [ … ] we do communicate well. We look at each other, like it is a good loving relationship, but I just, I just couldn’t see it.” (P14).

The positive cycle towards self-confidence and bonding brought into focus the direct impact participants had on their babies. Participants were able to acknowledge the importance of looking after themselves to look after their babies:

“Going to regular classes has been really good for me in terms of remembering my pre-mother identity and like connecting in with that just physically moving my body and seeing my body come back to health and vitality cause I really felt like I lost it in pregnancy and all of that connection and plus the good stuff that comes with just exercise and identify has been really positive in my like just looking after my mental wellbeing and gives me more capacity I think to bond with [sub baby]” (P16).

The importance of looking after one’s own mental wellbeing was described by P12 who expressed the importance of coregulation:

“I’ve noticed when he’s crying, if I’m agitated, he just cries more because he senses that, whereas if I go out really calm, low tone, like he just he, he will calm down quite quickly. Because I’ve got thinking I need to be able to regulate, to regulate me and regulate him. Can’t expect him just to regulate himself, he’s two years old. So when I’m fully aware of that, but I then I feel good for it because I know I’m in a- I’ve done that myself” (P12).

Highlighting the importance of trust, one participant who felt a trust in her subsequent foetus’s viability throughout pregnancy reported less anxiety related to the possibility of losing her baby and did not experience many challenges bonding:

“I just sort of had this feeling that she’s sort of robust for a baby (laughs). Yeah so there was sort of, I guess in terms of like the bond- my feeling towards her is that she’s quite strong and that we can work as a team and that I can (.) trust in her as long as I’m doing my bit” (P16).

#### 3.2.2 Theme 2: Navigating motherhood after perinatal bereavement with a subsequent baby

Theme 2, with its two subthemes *Making sense of the family story and Support as a scaffolding for bonding*, was characterised by participants’ attempts to understand their dual identity of being a mother to both a lost and a living baby. Bonding appeared to be shaped indirectly through a reconstruction of the family narrative, and the mothers’ ability to emotionally hold both children in mind. Bonding was facilitated through relational experiences between the mother, their lost baby and their subsequent baby.

##### 3.2.2.1 Subtheme 2.1: Making sense of the family story

Processing grief from previous loss was described to be inhibited by how society in general failed to acknowledge motherhood following loss:

“Some people didn’t think I was a mum. Because I hadn’t brought one home. That really hurt” (P15).

Accounts revealed attempts by participants to make sense of themselves as a mother to a lost and a living baby:

“Your identity after loss is that you’re a mum to a baby that isn’t here so (.) [subsequent baby] being my second child but my first living child. [ … ] my first experience of what a true parent is meant to look like in society” (P12).

While having to make sense of what it means to be a mother to a lost and a living baby, participants also reflected on having to navigate their family narrative. One area of emphasis related to their subsequent baby having a distinct identity, separate from their older sibling. Participants who touched upon this element did not consider their subsequent baby as a replacement baby; rather, they understood them as an additional child with their own distinct place in the family narrative. Some participants had direct experience of other people, including those close to them as well as healthcare professionals, hinting at their subsequent baby being a replacement, or an antidote to their grief, which was experienced by them as inconsiderate to the baby who they had lost. Instead, participants expressed feeling comfort when there was an acknowledgment of both babies.

Moving through the process of understanding the family narrative appeared to influence bonding experiences with their subsequent baby. Participants recognised that if they had not experienced loss, their subsequent baby might not have been part of their family story due to only wanting a certain number of children, or because of the timings of each pregnancy and how having the two babies in the timeframe would not have been physically possible (e.g., becoming pregnant before the due date of their baby who died). Attempts to make sense of their family narrative was accompanied by mixed emotions when trying to bond and form connections with their subsequent baby:

“Especially in the first few months after [subsequent baby] being born, I felt like every time I was like, “oh baby, I’m so glad you’re here”. I was almost saying like, ‘oh, I’m glad it happened to you [lost baby] because I wouldn’t have [subsequent baby]’. [ … ]. And I really struggled with that in terms of my bond because I almost felt guilty for being too bonded with [subsequent baby] because I felt like I was like doing [lost baby] a disservice then. And I’d say it was that with the bond that I massively struggle with, like couldn’t bond through pregnancy whatsoever, but then really struggle with almost what it meant for [lost baby] if I had a good bond with [subsequent baby]” (P19).

Most participants reflected on how they were navigating the relationship between their children. Although participants acknowledged the importance of recognising their lost baby as a member of the family, and importantly, a sibling to their subsequent baby, often participants expressed feeling ambivalent about what the “right” thing to do was when wanting to involve their lost baby in the family narrative with their subsequent baby. Anticipation regarding how this ongoing process would be managed in the future as their subsequent baby grows older was shared:

“I really do want to share with her about [lost baby]. But it’s just like at what age is that appropriate? ‘cause obviously like just death in general to children is a massive concept and like we obviously don’t want to scare her in any way” (P4).

Bonding with their subsequent baby appeared to be also shaped by participants’ experiences of coming to terms with their family narrative. Once participants were able to come to terms with their understanding of their family story, some participants were able to form connections with their subsequent baby, without the accompanying mixed emotions:

“I can settle knowing that [lost baby] is still a part of our family and has her place, helps me to actually say that [subsequent baby] is also part of our family and has his place, and I can love him just as much for who he is and [lost baby] can be who she is as well” (P11).

Some participants shared how feeling disconnected from the baby they lost was perceived to be related to their emotional closeness and connection to their subsequent baby. As a response, participants learnt to involve their subsequent baby in commemorating the life of the baby they had lost. Integrating the grieving process into shared experiences with the subsequent child appeared to facilitate the mother-infant bond with *both* babies. Creating ongoing connections between siblings helped participants to ensure both the baby that they lost and subsequent baby had a distinct place in the family narrative. The outcome of this appeared to be three-fold. Firstly, it seemed to allow mothers to continue to grieve for their loss and bond with their baby who passed away. Secondly, as mothers expressed feeling able to process their grief, they appeared to be more able to emotionally connect with their subsequent baby and thus, strengthen the mother-baby bond. Finally, connections were also made *between* siblings which helped participants complete the family narrative.

Each participant had a different way of forming connections between the siblings, such as giving all siblings

“the same middle name, just so that they’ve all got like a link to each other” (P9),

handing down items between siblings (such as teddys and clothes to follow the same traditions as they would if both babies were alive) or creating shared experiences between the lost and living baby, as explained by P4 and P12:

That was like really, really, really important for us to be able to have the same exact experience [ … ] to like bond with [subsequent baby] for a few days in the hospital the same way we bonded with [lost baby] in the hospital” (P4). “That’s where I chose to have [subsequent baby] because despite there being a lot of trauma at that hospital I felt that’s [lost baby]’s home” (P12).

The process of forming connections between siblings was summarised in the experience described by P3:

“I bought a bench by the lake where we live. And I take [subsequent baby] there, feed the ducks. And it’s got [lost baby]’s name on the bench. I take her there all the time to be honest. And I tell her “This is [lost baby]’s bench. This is your brother’s bench”. And I probably sound like a crazy person but one of my memories right before [lost baby] was born, the lake was frozen and all the ducks where on the top of the lake. And I remember being like, “I can’t wait to show you these ducks” and I was like talking to bump. And I’ve kind of carried that on where I’m like they’re [lost baby]’s ducks, they’re not his ducks. But when I take [subsequent baby] there, that’s what I tell her [ … ] at first, I felt like every time I said something about [lost baby] it was sad. And now I can say stuff about him and [subsequent baby]. And it’s like, this is my family. These are my babies. And part of it is always going to be sad because [lost baby] is not here. But it’s not completely overwhelmed with sadness because I can I can bring him into a part of something that is what we’re doing” (P3).

##### 3.2.2.2 Subtheme 2.2: Support as a scaffolding for bonding

Engagement with both perinatal bereavement networks throughout different stages of their experiences featured prominently across accounts, which revealed how accessing support from both peers and mental health professionals appeared to provide a framework to make sense of their complex loss experiences. The framework seemed to allow participants to further develop their family narrative and, thus, ability to bond with their subsequent baby.

Narratives revealed the different experiences of engaging with loss specific networks, compared to general social interactions with others. One key feature across accounts was related to questions regarding the family composition, such as

“how many children do you have?” and “is this your first [baby]? (P17)”.

Participants balanced wanting to answer truthfully, acknowledging their baby who died whilst also managing invalidating responses from others as expressed by P3:

“But then what I also hate when they go ‘oh sorry shouldn’t have asked you bye’. That’s hard again then cause I’m like ‘no you can ask me, you should now ask me what his name is’” (P3).

However, participants also expressed feeling a pressure to protect others from social discomfort that they might have experienced when loss was spoken about openly. Participants wanted to protect themselves emotionally from the awkwardness this might have brought up for others:

“It was also the starting school thing which was like getting to know people and being like “oh so, has [subsequent baby] got any brothers or sisters?” And I’m just like [sigh] “Why? Why just ask that question?” Because- I just tell people. Because I don’t like to deny her existence almost. So that’s really important to me. But it’s also really complicated because I guess the longer that we get away from it, people are like ‘why are you telling me that?’ ‘Cause there was nothing wrong with her. She should be here that’s like, that’s why” (P6).

Participants were able to find emotional safety by accessing peer support and meeting with others who shared their experience:

“sometimes you don’t even need to talk about it, there’s a lightness associated with being in a space with other people who experienced it because they know, they’ve got a better sense of what it’s like” (P6).

The *lightness* described here by P6 could be understood as a reduction in their felt sense of awkwardness related to attempts to manage social interactions with others. A shared knowing and understanding of what other parents have been through appeared fundamental to peer support networks:

“I know that she actually knew these things as opposed to like she hadn’t just read it in a textbook in a way like she she lived this experience. So that was, that was like invaluable” (P19).

Participants seemed to not have to carry the emotional labour inherent in non-loss specific social situations, creating a safe space in which participants felt comfortable to share as little or as much about their experience. A reduction in emotional labour appeared pivotal in allowing participants a space to process their grief, continue bonds with their lost baby whilst having more emotional space to connect with their subsequent baby.

Many participants also sought professional support for mental health difficulties, such as grief and post-traumatic stress, throughout their journey and some accessed specialist bonding support. Psychological therapy seemed to enable participants to take a concept that felt intangible, such as the idea of having a live baby or bonding and make it tangible. The core focus of psychological support did not emphasise *how* mothers could improve their bond but created an environment in which participants could witness their own mother-infant connection. Through therapy, some participants were guided to intentionally notice their bond with their subsequent baby which enabled them to

“see it … to believe it” (P14).

By doing so, participants expressed feeling reassured in their abilities to bond with their subsequent baby. For some participants, skills learnt appeared meaningful, taken beyond the therapy room and applied to their everyday lives:

“[After their first Video Interactive Guidance (VIG) therapy session] I literally broke down in tears, and I was just like ‘Wow! Like I don’t, I don’t see that. But I see it now like through you [therapist] showing me and explaining to me’ and it just that I think that was a huge turning point of, you know, realisation of I am doing OK. Mine and [subsequent baby’s] bond like it is there” (P14).

Support preferences reflected a wider pattern across accounts highlighting the need for trauma-informed, individualised care in creating a safe space for mothers to seek support. Acknowledgment and understanding was illustrated through the adaptation of processes to ensure the mother feels listened to and cared for, especially when the participants were offered support strategies to aid developing the bond with their subsequent baby:

“Loads of people were like, why don’t you just have a bath and like, you know, and just sit with your bump and stuff. And like because I’m really scared of getting an infection, so I’m not having a bath. [ … ] That’s not going to work for me in my situation. So, we need to think of other things that I can do. [Therapist] was just really good at like rather than being like ‘well, this is the only option’ kind of thing of trying to think a bit more about how I can do it and also just being like if you can’t, it’s OK.” (P13).

## 4 Discussion

### 4.1 Summary of main findings

This study was the first of its kind to exclusively focus on mothers’ experiences of bonding with a baby born following a previous perinatal loss throughout pregnancy and following the arrival of a live baby. Two overarching themes, with five subthemes, were developed to capture participants’ experiences, *Bracing for Loss* and *Navigating Motherhood After Perinatal Bereavement with a Subsequent Baby*. Mothers expressed an initial worry about their subsequent pregnancy ending in another loss during the early days of pregnancy; however, the extent to which this worry impacted bonding experiences through pregnancy to the birth of a live baby varied. Both themes demonstrate participants’ experiences of carefully managing the balance between self-protection from further loss and developing connection with their subsequent baby. Theme 1, *Bracing for Loss*, captured how mothers’ bonding with their subsequent baby was shaped by a persistent sense of perceived threat, managed through self-preservation, heightened responsibility to protect the baby, and gradually developing trust and confidence in themselves and their baby. Theme 2, *Navigating Motherhood After Perinatal Bereavement with a Subsequent Baby*, captured how mothers made sense of their family story and maternal identity by emotionally holding both the baby who died and their subsequent baby in mind, with peer and professional support helping some mothers to process grief and recognise connection. The process of bonding was not linear and oscillated due to social factors such as support accessed and societal views of perinatal bereavement, thus, bonding with a subsequent child after loss was not a clearly defined experience.

### 4.2 Interpretation

The present study advances existing literature by moving beyond stillbirth-focused research (30–32) to explore how a broader range of perinatal bereavement experiences may also influence how a woman experiences subsequent mother-infant bonding. The themes suggest that bonding is less influenced by the type of perinatal bereavement, but more from the way perinatal bereavement reorganises a mother’s experiences and understanding of motherhood.

A key contribution of the findings is that they not only confirm that psychological difficulties can extend into subsequent pregnancies following a previous perinatal bereavement (19, 46), but they illustrate how ambivalence in subsequent pregnancies is carefully managed as a way of *bracing for loss* (20, 21). This could be explained in part by the theory of “*emotional cushioning”* whereby women emotionally distance themselves from their pregnancy to avoid future hurt (20, 22). In the current study, participants shared experiences related to emotional cushioning, however, there was also evidence of participants choosing to actively engage and form connections with their foetus, which could be viewed as a contrast to the characteristics of emotional cushioning (i.e., avoiding emotional connection; 22). This distinction is important because both emotional cushioning and the intentional attempts at bonding appeared to serve the same purpose (e.g., to protect themselves from the potential of another loss) but were experienced in contrast, highlighting how different women may adopt different coping strategies to manage their worries. Thus, findings demonstrated how ways of managing emotional ambivalence in subsequent pregnancies were not universal.

Participants described how they navigated motherhood to a subsequent baby after a previous perinatal bereavement through making sense of their family narrative. Whilst connections had formed with their lost baby, mothers appeared to experience an increase in their capacity to bond with their subsequent baby. Rather than illustrating a disruption or delay to the development of the mother-infant bonding, the findings suggest that bonding is a dynamic and relational process influenced by several factors including previous trauma and a mother’s understanding of motherhood. Findings can be understood in line with literature identifying how perinatal bereavement can impact a mother’s maternal identity, while purposely forming lasting connections with their baby who died can support mothers to reconstruct their identity following loss (31, 47, 48). Findings indicated how accessing peer and professional support provided participants with an emotional scaffold that aided their ability to understand their family narrative which ultimately strengthened the development of their mother-infant bond. These findings build upon previous work identifying the important aspects of bereavement care including recognising the personhood of the baby and access to peer support networks in processing experiences of perinatal bereavement (49–52). Notably, a novel finding of the current study focused on how continuing bonds between a mother and their baby who died not only appeared to allow mothers to process their grief but also appeared to support maternal identity and be associated with mothers having an increase capacity to form an emotional connection to their subsequent child. This has been previous recognised in the work of Budak et al. (31) who discussed the importance of continuing bonds in aiding a mother’s grieving process after a previous stillbirth. Taken together, these findings indicate that the focus should not be on whether or not mothers are able to bond with subsequent children but how bonding becomes possible after loss. However, it should be acknowledged that many mothers did not receive formal psychosocial or psychological support to bond with their subsequent baby, and hence the exploration of support remained somewhat limited.

This study presents several clinical implications for healthcare services supporting mothers to develop bonds with a baby born after a previous perinatal bereavement.

Based on the present findings, services could recognise that bonding with a subsequent baby after perinatal bereavement may be shaped by mothers’ experiences of perceived threat. Across participants’ accounts, an initial apprehension that another loss could occur was a shared feature, although the extent and persistence of this worry varied. This suggests that normalising and validating emotional reactions during subsequent pregnancies after a loss, particularly worries about recurrence and self-preservation strategies, may be helpful. Participants also described how anxiety, emotional regulation and confidence in their parenting abilities shaped their capacity to bond. Therefore, clinical support could attend not only to the mother-infant bond itself, but also to mothers’ wider emotional wellbeing, their ability to regulate fear, and their confidence in caring for their subsequent baby. In practice, this may involve clinical psychologists and psychotherapies in clinical practice supporting mothers during pregnancy and after birth to notice moments of connection with their baby, understand fluctuations in bonding as understandable rather than problematic, and reduce the sense that they are solely responsible for ensuring their baby’s survival.

The current findings also suggest that, for some mothers, continuing bonds with the baby who died supported grief integration, maternal identity and emotional capacity to connect with their subsequent baby. This implication is grounded in participants’ accounts of making sense of their family narrative and creating ongoing connections between their lost and living babies. The wider literature offers further support for this interpretation. For example, Payne et al. (53) theorised that memory-making after the death of a baby can support parents’ grief processing and parental identity. The present study extends this by suggesting that continuing bonds may also remain clinically relevant in the longer term, including during subsequent pregnancy and parenting. Therefore, when appropriate and led by the mother’s preferences, services may consider supporting mothers to reflect on memory-making activities, acknowledge the ongoing place of the baby who died, and integrate these continuing bonds into therapeutic work. For mothers who continue to experience bonding difficulties with their subsequent baby, evidence-based relational interventions, such as Video Interactive Guidance (VIG; 54, 55), may also be helpful in enabling mothers to notice and strengthen existing moments of connection.

Alongside study-derived implications, the wider clinical literature points to the importance of compassionate, relational and trauma-informed psychological approaches tailored to individual needs. Acknowledging the impact of a previous loss on later experiences, particularly during subsequent pregnancies, is central to this approach. For example, psychological approaches that have been used and adapted to support perinatal populations are Cognitive Behavioural Therapy (CBT) and Compassion Focused Therapy (CFT). Strategies commonly used in CBT, such as mindfulness and cognitive restructuring, may help to alleviate worry during subsequent pregnancies; however, these techniques should not be applied prescriptively. Wenzel (56, 57) highlighted that asking mothers to evaluate evidence for and against feared outcomes may feel invalidating when the feared event has previously occurred. Instead, clinicians could support mothers to develop more compassionate appraisals of their worries and coping responses. Furthermore, emerging evidence suggests that compassion focused interventions including Perinatal Compassion Focused Therapy (P-CFT; 58), which is used across some UK perinatal mental health services, may be useful in clinical perinatal populations (59, 60). Future research would benefit from studies evaluating if interventions that support self-compassion and emotional regulation also have indirect benefits for mother-infant bonding.

Taken together, the findings from the present study indicate that support following perinatal bereavement should be individualised rather than prescriptive. Participants’ experiences varied in relation to worry, self-preservation, confidence, grief, family narrative and bonding; therefore, a single intervention approach is unlikely to meet all needs. Clinical psychologists and other perinatal practitioners may have an important role in completing comprehensive assessments and developing individualised formulations that distinguish between difficulties primarily related to trauma, grief, emotional regulation, parental confidence, or the mother-infant relationship. Such formulations could help ensure that support is responsive to both the mother’s needs and the developing relationship with her baby.

### 4.3 Strengths, limitations, and future directions

The present study had several methodological strengths. Diverse recruitment strategies were used to ensure a wide variety of experiences were captured, although most mothers were recruited via social media. Whilst most interviews took place online, participants were given the choice of in person or online interviews, increasing the accessibility of taking part in the research. Being flexible with the data collection procedures also increased the reach of the study geographically outside of the study location (Greater Manchester) and meant that potential participants in different areas of the UK could be involved, including other areas in the North-West, the Midlands and the South of England. Participants had a diverse range of perinatal bereavement and bonding experiences which added depth to the data and allowed nuances to be explored.

As NHS services are not accessible to all, particularly those from minoritised backgrounds (61), efforts were made to recruit a heterogeneous sample using social media (62) and third sector organisations (such as Sands (63)). However, whilst the experiences of participants were diverse, participant characteristics were largely homogenous with all participants being heterosexual and a vast majority being white and married which reflects a broader issue within the field of perinatal research and may limit the transferability of the findings. It would be of importance for future studies to recruit women from minoritised ethnicity and/or sexuality backgrounds, using purposeful sampling. It may also be interesting to explore the potential impact of particular types of losses, comparable to Blocksidge et al.’s (32) IPA study of stillbirth, as part of a future study. Finally, as this was the first study to exclusively explore mother-infant bonding following a previous perinatal bereavement, the goal was to recruit a broad range of bonding experiences. Not all the participants in the study reported difficulties bonding with their subsequent babies. For in-depth understanding into the challenges of bonding following a previous perinatal bereavement to be developed, it would be helpful for future studies to focus on women who report clinically significant bonding difficulties. Focusing on difficulties experienced would allow mental health services that aim at supporting mothers to develop the mother-infant bond (such as perinatal mental health teams and children and parenting services) to understand the difficulties experienced by those who are more likely to present to their services.

### 4.4 Conclusions

The present qualitative study demonstrates that previous perinatal bereavement profoundly shaped mothers’ experiences of bonding with a subsequent baby. Rather than representing a straightforward transition from loss to new motherhood, bonding emerged as a complex and evolving process in which mothers moved from anticipating and fearing another loss towards learning to live with, and emotionally invest in, a subsequent live baby. This process involved negotiating the enduring presence of bereavement alongside the developing relationship with the new baby, while gradually coming to terms with the family’s story of loss and survival. Crucially, mothers’ capacity to develop and sustain a bond with their subsequent baby was supported by access to meaningful peer and professional networks, which provided opportunities for their experiences to be recognised, understood and validated. Taken together, these findings highlight that bonding following perinatal bereavement is not simply a matter of establishing a relationship with a new baby; it involves integrating past loss into an evolving maternal and family identity. Recognition of this complexity is important for ensuring that mothers are not expected to experience a subsequent birth as an uncomplicated new beginning, but are instead offered support that acknowledges both the continuing significance of their loss and the emerging relationship with their subsequent child.

## Data Availability

The datasets presented in this article are not readily available to protect participant confidentiality. Requests to access the datasets should be directed the corresponding author.

## Footnotes

1 Note: This time period was chosen to be in line with the service’s clinical criteria for accepting referrals.

## References

[1] Donegan G Noonan M Bradshaw C . Parents experiences of pregnancy following perinatal bereavement. Midwifery. (2023) 121:103673. doi: 10.1016/j.midw.2023.103673 37037073

[2] Blencowe H Hug L Moller A You D Moran AC . Definitions, terminology and standards for reporting of births and deaths in the perinatal period: International Classification of Diseases (ICD‐11). Int J Gynecology Obstetrics. (2024) 168:1–9. doi: 10.1002/ijgo.15794 39127912 PMC11649847

[3] Tommy’s . Baby Loss Information and Support; Types of Miscarriage (2026). Available online at: https://www.tommys.org/baby-loss-support/miscarriage/types-miscarriage (Accessed August 12, 2026).

[4] National Institute for Health and Care Excellence . Ectopic Pregnancy and Miscarriage: Diagnosis and Initial Management (2026). Available online at: https://www.nice.org.uk/guidance/ng126 (Accessed August 12, 2026). 31393678

[5] Tommy’s . Molar Pregnancy: Definition, Types, Symptoms and Risk Factors (2024). Available online at: https://www.tommys.org/baby-loss-support/molar-pregnancy-information-support (Accessed August 12, 2026).

[6] National Institute for Health and Care Excellence (NICE) . Abortion Care (2025). Available online at: https://www.nice.org.uk/guidance/ng140 (Accessed August 12, 2026).

[7] Tommy’s . Baby Loss Information and Support; Terminating a Pregnancy for Medical Reasons (2026). Available online at: https://www.tommys.org/baby-loss-support/tfmr-terminating-pregnancy-medical-reasons (Accessed August 12, 2026).

[8] Felker A Patel R Kotnis R Kenyon S Knight M . Saving Lives, Improving Mothers’ Care: Lessons Learned to Inform Maternity Care From the UK and Ireland Confidential Enquiries Into Maternal Deaths and Morbidity 2021–23 (2025). National Perinatal Epidemiology Unit, University of Oxford. Available online at: https://www.npeu.ox.ac.uk/mbrrace-uk/reports/maternal-reports/maternal-report-2021-2023 (Accessed August 12, 2026).

[9] Office for National Statistics (ONS) . Prenatal Deaths in England and Wales, 2018 to 2022 (2023). Available online at: https://www.ons.gov.uk/aboutus/transparencyandgovernance/freedomofinformationfoi/prenataldeathsinenglandandwales2018to2022 (Accessed August 12, 2026).

[10] Heazell AEP Siassakos D Blencowe H Burden C Bhutta ZA Cacciatore J . Stillbirths: economic and psychosocial consequences. Lancet. (2016) 387:604–16. doi: 10.1016/S0140-6736(15)00836-3 26794073

[11] Quenby S Gallos ID Dhillon-Smith RK Podesek M Stephenson MD Fisher J . Miscarriage matters. Lancet. (2021) 397:1658–67. doi: 10.1016/s0140-6736(21)00682-6 33915094

[12] World Health Organization . Stillbirths (2023). Available online at: https://www.who.int/health-topics/stillbirth#tab=tab_1 (Accessed March 14, 2026).

[13] World Health Organization . Newborn Mortality (2024). Available online at: https://www.who.int/news-room/fact-sheets/detail/newborn-mortality (Accessed March 14, 2026).

[14] Burden C Bradley S Storey C Ellis A Heazell AEP Downe S . From grief, guilt pain and stigma to hope and pride – a systematic review and meta-analysis of mixed-method research of the psychosocial impact of stillbirth. BMC Pregnancy and Childbirth. (2016) 16(1). doi: 10.1186/s12884-016-0800-8 PMC4719709 26785915

[15] Herbert D Young K Pietrusińska M MacBeth A . The mental health impact of perinatal bereavement. J Affect Disord. (2022) 297:118–29. doi: 10.1016/j.jad.2021.10.026 34678403

[16] Berry SN . The trauma of perinatal bereavement: A scoping review. Trauma Care. (2022) 2:392–407. doi: 10.3390/traumacare203003230654563

[17] Zhang X Chen Y Zhao M Yuan M Zeng T Wu M . Complicated grief following the perinatal bereavement. BMC Pregnancy Childbirth. (2024) 24:772. doi: 10.1186/s12884-024-06986-y 39578811 PMC11583632

[18] Lamont K Scott NW Jones GT Bhattacharya S . Risk of recurrent stillbirth. BMJ. (2015) 350:h3080. doi: 10.1136/bmj.h3080 26109551

[19] Mills T Ricklesford C Cooke A Heazell A Whitworth M Lavender T . Parents’ experiences and expectations of care. BJOG. (2014) 121:943–50. doi: 10.1111/1471-0528.12656 24589119

[20] Côté-Arsenault D Marshall R . One foot in - one foot out. Res Nurs Health. (2000) 23:473–85. doi: 10.1002/1098-240x(200012)23:6%3C473::aid-nur6%3E3.0.co;2-i 10.1002/1098-240X(200012)23:6<473::AID-NUR6>3.0.CO;2-I 11130606

[21] Meredith P Wilson T Branjerdporn G Strong J Desha L . Not just a normal mum: a qualitative investigation of a support service for women who are pregnant subsequent to perinatal bereavement. BMC Pregnancy Childbirth. (2017) 17:1–12. doi: 10.1186/s12884-016-1200-9 28056861 PMC5217635

[22] Côté‐Arsenault D Donato K . Emotional cushioning in pregnancy after perinatal bereavement. J Reprod Infant Psychol. (2011) 29:81–92. doi: 10.1080/02646838.2010.51311537339054

[23] Lee L McKenzie-McHarg K Horsch A . The impact of miscarriage and stillbirth. J Reprod Infant Psychol. (2016) 35:32–52. doi: 10.1080/02646838.2016.1239249 29517293

[24] Kinsey CB Hupcey JE . State of the science of maternal–infant bonding. Midwifery. (2013) 29:1314–20. doi: 10.1016/j.midw.2012.12.019 23452661 PMC3838467

[25] Bas GL Youssef G Macdonald JA Teague S Mattick R Honan I . The role of antenatal and postnatal maternal bonding in infant development. J Am Acad Child Adolesc Psychiatry. (2022) 61:820–829.e1. doi: 10.1016/j.jaac.2021.08.024 34555489

[26] Winston R Chicot R . The importance of early bonding. J Primary Care. (2016) 8:12–4. doi: 10.1080/17571472.2015.1133012 28250823 PMC5330336

[27] O’Dea GA Youssef GJ Hagg LJ Francis LM Spry EA Rossen L . Associations between maternal psychological distress. Arch Women’s Ment Health. (2023) 26:441–52. doi: 10.1007/s00737-023-01332-1 37316760 PMC10333415

[28] Shorey S Asurlekar AR Chua JS Lim LHK . Influence of oxytocin on parenting behaviours. Dev Psychobiol. (2022) 65:e22359. doi: 10.1002/dev.22359 36811366

[29] Vagos P Mateus V Silva J Araújo V Xavier A Palmeira L . Mother–infant bonding. J Reprod Infant Psychol. (2023) 43:472–86. doi: 10.1080/02646838.2023.2242379 37525320

[30] Campbell-Jackson L Bezance J Horsch A . A renewed sense of purpose. BMC Pregnancy Childbirth. (2014) 14:1–12. doi: 10.1186/s12884-014-0423-x 25522655 PMC4279693

[31] Budak AMÜ Larkin M Harris G Blissett J . Mothers’ accounts of their stillbirth experiences. BMC Pregnancy Childbirth. (2015) 15:1–11. doi: 10.1186/s12884-015-0700-3 26463456 PMC4604712

[32] Blocksidge H Heazell AEP Wittkowski A Smith DM . The sorrow comes when I’m having moments of joy—experiences of parenting a live baby following a previous stillbirth: an interpretative phenomenological analysis. Frontiers in Psychology. (2024) 15. doi: 10.3389/fpsyg.2024.1485278 PMC11580424 39575342

[33] Braun V Clarke V . Using thematic analysis in psychology. Qual Res Psychol. (2006) 3:77–101. doi: 10.1191/1478088706qp063oa

[34] Braun V Clarke V . Reflecting on reflexive thematic analysis. Qual Res Sport Exercise Health. (2019) 11:589–97. doi: 10.1080/2159676x.2019.162880637339054

[35] Tong A Sainsbury P Craig J . Consolidated criteria for reporting qualitative research (COREQ): a 32-item checklist for interviews and focus groups. Int J For Qual Health Care. (2007) 19:349–57. doi: 10.1093/intqhc/mzm042 17872937

[36] Health Research Authority. Public involvement in health and social care research. (2021) Available online at: https://www.hra.nhs.uk/planning-and-improving-research/best-practice/public-involvement/ (Accessed April 07, 2026).

[37] Malterud K Siersma VD Guassora AD . Sample size in qualitative interview studies: Guided by information power. Qual Health Res. (2016) 26:1753–60. doi: 10.1177/1049732315617444 26613970

[38] Guest G Bunce A Johnson L . How many interviews are enough? An experiment with data saturation and variability. Field Methods. (2006) 18:59–82. doi: 10.1177/1525822x05279903

[39] Braun V Clarke V . Can I use TA? Should I use TA? Should I not use TA? Counselling Psychother Res. (2020) 21:37–47. doi: 10.1002/capr.1236042587375

[40] Braun V Clarke V . Thematic analysis: A practical guide. London: SAGE. (2021).

[41] Gough B Madill A . Subjectivity in psychological science. psychol Methods. (2012) 17:374–84. doi: 10.1037/a0029313 22799622

[42] Braun V Clarke V . Conceptual and design thinking for thematic analysis. Qual Psychol. (2021) 9:3–26. doi: 10.1037/qup000019627371692

[43] Dwyer SC Buckle JL . The space between. Int J Qual Methods. (2009) 8:54–63. doi: 10.1177/160940690900800105

[44] Fletcher AJ . Applying critical realism in qualitative research. Int J Soc Res Method. (2017) 20:181–94. doi: 10.1080/13645579.2016.114440137339054

[45] Alderson P . Critical Realism for Health and Illness Research: A Practical Introduction. Bristol: Bristol University Press (2021).

[46] Hunter A Tussis L MacBeth A . The presence of anxiety, depression and stress in women and their partners during pregnancies following perinatal loss: A meta-analysis. J. Affect. Disord. (2017) 223:153–64. doi: 10.1016/j.jad.2017.07.004 28755623

[47] Beato BVG Machado-Kayzuka GC Neris RR Payne E de Andrade Alvarenga W Leite ACAB . (2025). Experiences and long-term repercussions of perinatal grief in women after perinatal bereavement: a meta-ethnography. Frontiers in Psychiatry. 16. doi: 10.3389/fpsyt.2025.1661483 PMC12754219 41480336

[48] Rossen L Opie JE O’Dea Gypsy . A Mother’s Voice: The Construction of Maternal Identity Following Perinatal Loss. OMEGA - Journal of Death and Dying. (2023). doi: 10.1177/00302228231209769 PMC12769918 37871980

[49] Berry SN . The Trauma of Perinatal Loss: A Scoping Review. Trauma Care. (2022) 2(3):392–407. doi: 10.3390/traumacare2030032

[50] Farrales LL Cacciatore J Jonas-Simpson C Dharamsi S Ascher J Klein MC . What bereaved parents want health care providers to know when their babies are stillborn: a community-based participatory study. BMC Psychol. (2020) 8:1–9. doi: 10.1186/s40359-020-0385-x 32066494 PMC7027220

[51] Nuzum D Meaney S O’Donoghue K . The impact of stillbirth on bereaved parents: A qualitative study. PloS One. (2018) 13:e0191635. doi: 10.1371/journal.pone.0191635 29364937 PMC5783401

[52] Thomson G McNally L Nowland R . Experiences and impacts of psychological support following adverse neonatal experiences or perinatal bereavement: a qualitative analysis. BMC Pregnancy Childbirth. (2024) 24. doi: 10.1186/s12884-024-06713-7 39215235 PMC11365156

[53] Payne E Silverio SA Fellows RE Heywood LE Burgess K Storey C . From mourning to memorialising. Women Birth. (2025) 38:101902. doi: 10.1016/j.wombi.2025.101902 40315808

[54] National Institute for Health and Care Excellence [NICE] . Social and Emotional Wellbeing: Early Years (PH40) (2012). Available online at: https://www.nice.org.uk/guidance/ph40 (Accessed March 14, 2026).

[55] National Institute for Health and Care Excellence . Children’s Attachment (NG26) (2015). Available online at: https://www.nice.org.uk/guidance/ng26 (Accessed March 14, 2026).

[56] Wenzel A . Cognitive Behavioural Therapy for Perinatal Distress. London: Routledge (2015).

[57] Wenzel A . Cognitive behavioural therapy for pregnancy loss. Psychotherapy. (2017) 54:400–5. doi: 10.1037/pst0000132 29251960

[58] Cree M . Compassion focused therapy. Int J Cogn Ther. (2010) 3:159–71. doi: 10.1521/ijct.2010.3.2.15937804533

[59] Millard LA Wittkowski A . Compassion focused therapy for women. Front Psychiatry. (2023) 14:1288797. doi: 10.3389/fpsyt.2023.1288797 38179247 PMC10766363

[60] Millard-Brewer LA Wittkowski A . Assessing the effects of group perinatal compassion focused therapy. Front Psychol. (2026) 17:1800377. doi: 10.3389/fpsyg.2026.1800377 41969876 PMC13062174

[61] National Health Service (NHS) Race and Health Observatory . Ethnic Inequalities in Improving Access to Psychological Therapies (IAPT) (2023). Available online at: https://www.nhsrho.org/wp-content/uploads/2023/10/Ethnic-Inequalities-in-Improving-Access-to-Psychological-Therapies-IAPT.Full-report.pdf (Accessed April 14, 2026).

[62] Kayrouz R Dear BF Karin E Titov N . Facebook as an effective recruitment strategy. Internet Interventions. (2016) 4:1–10. doi: 10.1016/j.invent.2016.01.001 30135786 PMC6096235

[63] Sands . Baby Loss and Pregnancy Loss Statistics (2025). Available online at: https://www.sands.org.uk/baby-loss-and-pregnancy-loss-statistics (Accessed March 14, 2026).

