# Supplementary material for: Mothers’ experiences of bonding with their subsequent child following a previous perinatal bereavement
Source: Front Psychiatry. 2026 Oct 7;17:1894993. doi: 10.3389/fpsyt.2026.1894993 (PMC13684786; doi:10.3389/fpsyt.2026.1894993)
Supplement: Supplementary file 2 [file Table1.docx]

**Supplementary materials: Interview guide**
